# Crosstalk between macrophages and astrocytes affects proliferation, reactive phenotype and inflammatory response, suggesting a role during reactive gliosis following spinal cord injury

**DOI:** 10.1186/s12974-015-0327-3

**Published:** 2015-05-30

**Authors:** Niels Haan, Bangfu Zhu, Jian Wang, Xiaoqing Wei, Bing Song

**Affiliations:** Cardiff Institute of Tissue Engineering & Repair, School of Dentistry, College of Biomedical and Life Sciences, Cardiff University, Heath Campus, Cardiff, CF14 4XY UK; Neuroscience and Mental Health Research Institute, College of Biomedical and Life Sciences, Cardiff University, Hadyn Ellis Building, Maindy Road, Cardiff, CF24 4HQ UK; Institute of Neurosciences, Fourth Military Medical University, 169 West Changle Road, Xi’an, 710032 China; Department of Dermatology, No. 1 Hospital of China Medical University, Shenyang, 110001 China

**Keywords:** Glia, Spinal cord, Traumatic injury, Immune response, Neuroinflammation

## Abstract

**Background:**

Large-scale macrophage infiltration and reactive astrogliosis are hallmarks of early spinal cord injury (SCI) pathology. The exact nature of the macrophage response and relationship between these phenomena have not been explored in detail. Here, we have investigated these responses using a combination of in vivo SCI models, organotypic and primary cultures.

**Methods:**

In vivo macrophage response was investigated using a contusive injury mouse model. Interactions between astrocytes and macrophages were studied in primary or organotypic cultures. Proliferation was assessed though MTT assay and nucleotide incorporation and gene expression changes through qPCR.

**Results:**

Seven days following contusive SCI, a mixed M1/M2 macrophage response was seen in the injury site. Conditioned medium from primary M1, but not M2, macrophages are able to induce astrocyte proliferation in both organotypic spinal cord cultures and primary astrocytes. Soluble factors from M1 macrophages induce a reactive astrocyte gene expression pattern, whereas M2 factors inhibit expression of these genes. M2-stimulated astrocytes are also able to decrease both M1 and M2 macrophage proliferation and decrease TNFα production in M1 macrophages.

**Conclusions:**

These results suggest a strong role of M1 macrophages in inducing reactive astrogliosis and the existence of an astrocyte-mediated negative feedback system in order to dampen the immune response. These results, combined with the poor outcomes of the current immunosuppressive steroid treatments in SCI, indicate the need for more targeted therapies, taking into account the significantly different effects of M1 and M2 macrophages, in order to optimise outcome.

**Electronic supplementary material:**

The online version of this article (doi:10.1186/s12974-015-0327-3) contains supplementary material, which is available to authorized users.

## Background

Spinal cord injury (SCI) is one of the leading causes for long-term disability in the Western world. Current treatments are limited to early administration of steroids, with initial studies suggesting good outcomes [1], but their actual efficacy has been debated [2, 3], and significant functional improvement is still difficult to achieve.

The use of steroids illustrates the large role the immune system plays in the pathology and resolution of SCI. Following SCI, the initial phagocytotic response is from the resident microglia, however, as the blood–brain barrier is generally compromised following SCI, peripheral macrophages rapidly infiltrate the spinal cord and become the predominant phagocytosing cell type at 3 days post injury (dpi) [4] in mouse models. Additionally, the infiltrating peripheral macrophages are responsible for the majority of secondary neuronal cell death in the subacute phase of SCI [5]. Indeed, complete depletion of the macrophage population following SCI led to functional improvement in one study [6].

However, the picture may not be that simple, as macrophages are not a monolithic cell population. Macrophages consist mainly of two subtypes, the pro-inflammatory M1 type and an anti-inflammatory/regenerative M2 type [7]. M2 macrophages have been implicated in tissue regeneration in muscle [8] and skin [9]. In an experimental demyelination model in the brain, M2 type macrophages were required for initiation of remyelination [10], and it has been suggested that M2 macrophages are neurosupportive in SCI as well [11]. This indicates a potential regenerative role for these cells in the nervous system. There have been several reports about the exact nature of the macrophage response in SCI, with varying proportions of M1 and M2 having been described [11–15]. How these macrophage phenotypical differences affect the development and resolution of SCI is still unclear.

In the longer term following SCI, the formation of the glial scar by reactive astrocytes is a crucial factor in the potential long-term recovery of functionality. Although it has recently been appreciated that at least some of the glial scar may be derived from pericytes [16], at its heart, the glial scar is comprised of a dense network of fibrous astrocytes, filling in and surrounding the injury site. The presence of large quantities of inhibitory extracellular proteoglycans in the scar restricts the regrowth of endogenous axons into the injury area [17]. Although traditionally viewed as detrimental to regeneration, the reactive astrocytes of the glial scar may also have beneficial effects, including aiding in the repair of the blood–brain barrier and modulation of the immune cell response [18–20].

Although the glial scar is well studied, the initiation of this astrocyte response is not well characterised. Factors known to increase glial scar formation in the spinal cord include TGFβ1 [21] and INFγ [1], amongst others. It has also been suggested that Fgf2 may be used to decrease scar formation and improve axonal permissibility [22], suggesting growth factors are important in glial scar development.

In short, the complete picture of scar induction and formation is far from clear. It is likely that the large-scale immune cell infiltration plays in important role in this. In this study, we aim to investigate the macrophage response to SCI and the interaction between macrophages and astrocytes to elucidate the role these play in the development of the glial scar. We report that M1 macrophages, present in significant numbers following SCI, are able to induce both astrocyte proliferation and a reactive phenotype, which can be partially counteracted by M2 macrophages. Additionally, astrocytes previously stimulated by M2 macrophages are able to decrease macrophage proliferation and activity, indicating an important role of the astrocyte-macrophage axis in SCI.

## Materials and methods

### Spinal cord impact model

All animal work was performed according to the procedures in the Animals (Scientific Procedures) Act 1986 and local guidelines. Healthy female C57Bl/6 J mice of 18–20 g in weight were subjected to a spinal cord impact. Under isofluorane anaesthesia, the dorsal skin was opened and a laminectomy was performed at the T9 level. For sham animals, at this point, the skin and muscle layers were closed and the animals were allowed to recover. In the impact group, animals were subjected to a controlled spinal impact of 50 kDyn using the Infinite Horizon Impactor device (Precision Scientific Instrumentation, IH-0400). Following recovery of both groups, functional impairment was assessed using the BBB scale [23]. Only animals with a BBB score of <3 were considered in the impact group and >18 in the sham group, with a minimum of four animals per group.

### Tissue harvesting and preparation

Seven days following SCI surgery, after the onset of glial scar formation, animals were sacrificed using CO_2_ exposure and transcardially perfused with PBS followed by 4 % PFA in PBS. Spinal cords were removed and postfixed in 4 % PFA in PBS overnight. They were then transferred to 30 % sucrose for cryoprotection for 2 days, before being mounted in OCT for sectioning in 15-μm sections.

### Immunohistochemistry

Sections were air-dried, postfixed in cold 1:1 acetone/methanol for 2 min and blocked in 2 % BSA and 1 % Triton X-100 in PBS for 1 h. Antibodies were applied overnight at 4 °C and were: anti-CD68 (Abcam, ab53444, 1:100), anti-arginase-1 (Santa Cruz Biotechnology, sc-18355, 1:50), anti-iNOS (BD Biosciences, 610329, 1:100) and anti-GFAP (Cell Signaling, 12389S, 1:250). Controls included species- and concentration-matched isotype controls, which showed no staining (data not shown). Antibodies were detected with the relevant secondary antibodies conjugated to Alexa 488 or 594 (Life Technologies, 1:500), sections were mounted in DAPI containing mounting medium and were imaged through Z-scanning on an Applied Precision DeltaVision microscope.

### Spinal cord organotypic cultures

Healthy C57BL/6 J mice of 6–12 weeks of age were sacrificed through CO_2_ exposure and the spinal cord removed. Meninges and other remaining tissue were dissected off, and 500-μm sections of the thoracic and abdominal cord were made using a McIlwain tissue chopper. Sections were then embedded in Matrigel (Corning, #354234) and cultured in DMEM/F12 medium containing 20 % FBS, 1 % penicillin/streptomycin (P/S) and 2 mM L-glutamine at 37 °C and 5 % CO_2_. For immunohistochemistry and EdU detection, organotypic cultures were fixed for 30 min in 4 % PFA in PBS, cryoprotected in 30 % sucrose overnight and sectioned in the same manner as the whole cords.

### Primary cell isolation and culture

Primary macrophages were cultured from bone marrow progenitors. Bone marrow was isolated from femurs by flushing the bone cavity with cold RPMI medium. Marrow was pelleted by centrifugation and erythrocytes were lysed using ammonium chloride lysing buffer (BD Pharmlyse, 55899). Following two washes in cold RPMI and straining through a 50-μm cell strainer, bone marrow progenitors were cultured in T75 flasks at 1 × 10^6^/ml in RPMI containing 10 % FBS, 2 mM L-glutamine and 1 % P/S. For differentiation into M1 macrophages, 20 ng/ml GM-CSF was added, for M2 macrophages, 20 ng/ml of both M-CSF and Il-4 were added, all from Peprotech, products 315-03A, 315-02B and 214-14A, respectively. Following 7 days of culture, macrophages were differentiated and ready for use.

Mouse primary astrocytes were isolated from neonatal mixed glia cultures as previously described for rat [24].

### Conditioned medium production and use

Conditioned media were prepared from M1 and M2 macrophages, as well as from astrocytes that were non-stimulated or stimulated with M1 or M2 macrophage conditioned medium. In cultures that were previously stimulated (with differentiation growth factors in the case of macrophages and conditioned medium in the case of astrocytes), cells underwent several medium changes over 24 h following stimulation to ensure none of the stimulating factors carried over into the conditioned medium. For all cell types, conditioned medium was prepared by incubating cells in their normal culture medium for 3 days. Following conditioning, medium was filtered through 0.22-μm syringe filters, aliquoted and stored at −80 °C until use.

For all conditioned medium experiments, cultures or organotypic cultures were simulated with 25 % conditioned medium for macrophage medium or 50 % for astrocyte medium, added to their usual culture medium. For astrocyte experiments, controls consisted of incubation with a matched percentage unconditioned medium of the same type as the conditioned medium. For macrophage experiments, controls consisted of conditioned medium from unstimulated astrocytes. All experiments were repeated using several batches of conditioned medium, from cells from different isolations, to avoid batch-specific effects.

### MTT assays

Cells were seeded in 96-well plates at 5 × 10^4^ cells/well, at 4–6 wells per condition and allowed to adhere overnight. The following day, the medium was changed to experimental conditions, and the cells were allowed to proliferate for 2, 4, or 6 days. MTT was then added to cultures to a 0.5 mg/ml working concentration in the medium from a 5-mg/ml stock solution in PBS. Following 1–3 h of incubation, medium was removed, precipitated dye was resuspended in DMSO and absorbance was measured at 570 nm. Absorbance was quantified as relative percentages compared to control conditions.

Organotypic slice cultures were incubated in experimental conditions for 72 h with an *n* of 4 per condition, before MTT was added at 0.5 mg/ml to the culture medium. Following 2–4 h of exposure, slice cultures were removed from the Matrigel into individual microcentrifuge tubes and dried under vacuum to obtain an accurate dry tissue weight. Tissue was then resuspended in 10 % SDS in H_2_O at 25 mg dry weight/ml and dissolved at 60 °C under agitation for 6 h. Following centrifugation to remove any non-dissolved tissue, absorbance was measured at 570 nm. Absorbance was quantified as relative percentages compared to control conditions.

### Nucleotide incorporation assays and detection

Cells were seeded at 1 × 10^5^ cells/well in poly-D-lysine coated 8-well chamberslides and treated identical to those used for MTT assays. Twenty-four hours prior to the end of the protocol, 10-μM EdU was added from a 100-mM 1:10 DMSO/H_2_O stock. For organotypic cultures, EdU was added 48 and 24 h prior to end of protocol. Cells were then fixed for 20 min in 4 % PFA in PBS, and EdU was detected as previously described [25]. Proliferation was determined as percentage EdU incorporating cells of total number of cells as determined by DAPI nuclear staining. Organotypic cultures were sectioned prior to detection of EdU. Proliferation was quantified as the number of EdU incorporating cells per square millimetre of section, with section area being determined in ImageJ. This area measure corrected for varying sizes or organotypic cultures.

### Quantitative PCR

RNA was extracted from unstimulated and M1- and M2-stimulated astrocytes (*n* = 3) using the RNeasy kit (Qiagen), according to manufacturer’s instructions. RNA was then reverse transcribed using M-MLV (Promega, M170A). Quantitative PCR for reactive astrocyte genes *GFAP*, *nestin*, *vimentin*, *cdk1*, *ccnb1*, *ccnd1*, *mki67*, *lcn2* and *serpinA3*, with *gapdh* as housekeeping, was performed on a Quantstudio 6 thermal cycler using PrimerDesign mastermix (PrecisionPLUS-LR-SY), using a 60 °C annealing temperature. Primers used are listed in Additional file 1: Table S1. Data was analysed using the ΔΔC_t_ method and expressed as fold changes from control samples.

### TNF production in macrophages and ELISA

M1 and M2 macrophages were stimulated with conditioned medium from astrocytes previously stimulated by M1- or M2-conditioned medium for 3 days. Following stimulation, macrophages were replated into 24-well plates at 2 × 10^5^ cells/well, activated with 1 μg/ml of β-glucan (Sigma Aldrich, C7821), and medium was collected after 48 h. TNF levels were determined using sandwich ELISA. Plates were coated with anti-TNF antibody (eBioscience, 14-7325-85, 1:500) overnight at 4 °C, blocked with blocking buffer (eBioscience 00-4202-55), and the samples were incubated overnight at 4 °C. Following binding of biotinylated anti-TNF antibody (eBioscience, 13-7326-85, 1:500) for 2 h at room temperature, streptavidin-HRP (eBioscience, 00-4106-93, 1:250) was added for 45 min at room temperature, followed by TMB substrate (eBioscience, 00-4201-56) for 20–30 min, before being read on a spectrophotometer at 450 nm.

### Statistical analysis

Groups were compared using one-way ANOVA with Tukey post hoc testing in SPSS 20. Data is expressed as average ± SEM.

## Results

### SCI induces a mixed M1/M2 macrophage response

**Fig. 1:**
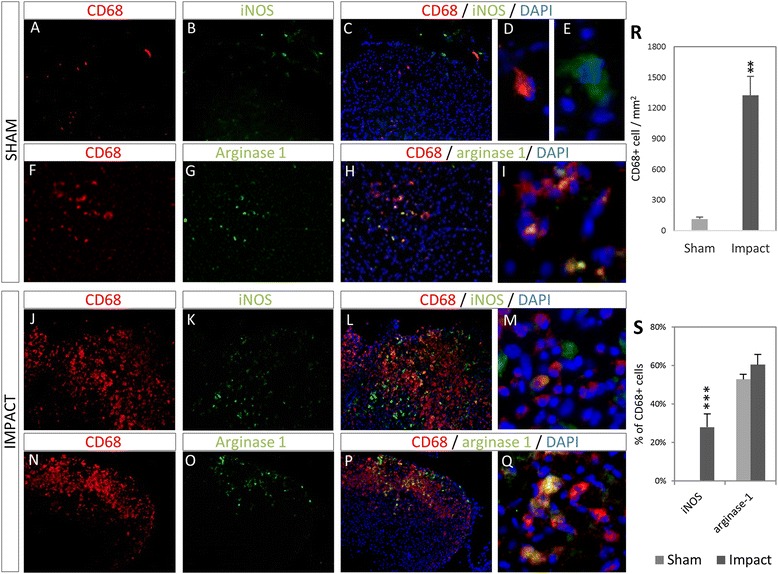
Macrophage response 7 days after contusive SCI. In sham surgical animals, low numbers of CD68+ cells are seen (**a**–**i**). These cells do not express the M1 marker iNOS (**b**, **c**), although there is iNOS expression in CD68− cells. The M2 marker arginase-1 is widely expressed in CD68+ cells (**f**–**i**). In contrast, following contusive injury, large number of macrophages is seen in the vicinity of the injury (**j**–**q**). iNOS expression is widespread, in both CD68+ and CD68− cells (**j**–**m**). As in the sham control, arginase is widely expressed (**n**–**q**). **r** The total number of CD68+ cells per square millimetre of section increased significantly in SCI compared to sham. **s** Quantification of macrophage subtypes following injury shows a constant fraction of arginase-1+/CD68+ cells in both sham and impact groups, whereas there is significant increase in number of iNOS+/CD68+ cells

Contusive injury was performed on mice to investigate the nature of the macrophage response in our injury model. BBB scores showed both successful sham and impact groups (*n* = 4 for both), with scores of 18.6 ± 0.8 and 0.9 ± 0.3, respectively. At 7 DPI, the macrophage response was investigated by immunohistochemistry for CD68 (pan-macrophage marker) and iNOS (M1 marker) and arginase-1 (M2 marker). As shown in Fig. 1, spinal cords from the sham group show very low numbers of CD68+ cells, with some iNOS expression, though not in the CD68+ cells. Arginase-1 expression is widespread, including in CD68+ cells. Following contusive injury, large numbers of CD68+ cells can be seen, with widespread expression of both iNOS and arginase-1 in CD68+ and CD68− cells. When quantified (Fig. 1r), the large increase in CD68+ cells following SCI is evident, 113 ± 19 vs 1324 ± 185 cells/mm^2^ (*p* = 0.002). Quantification of marker expression on CD68+ cells (Fig. 1s) shows that the percentage of CD68+ cells that express arginase remains largely unchanged (52.8 ± 6.9 % vs 60.5 ± 5.3 %), while the percentage of iNOS expressing cells is strongly increased (0 % vs 28.1 ± 2.8 %, *p* < 0.001).

### M1 macrophage soluble factors induce proliferation in spinal cord organotypic cultures

**Fig. 2:**
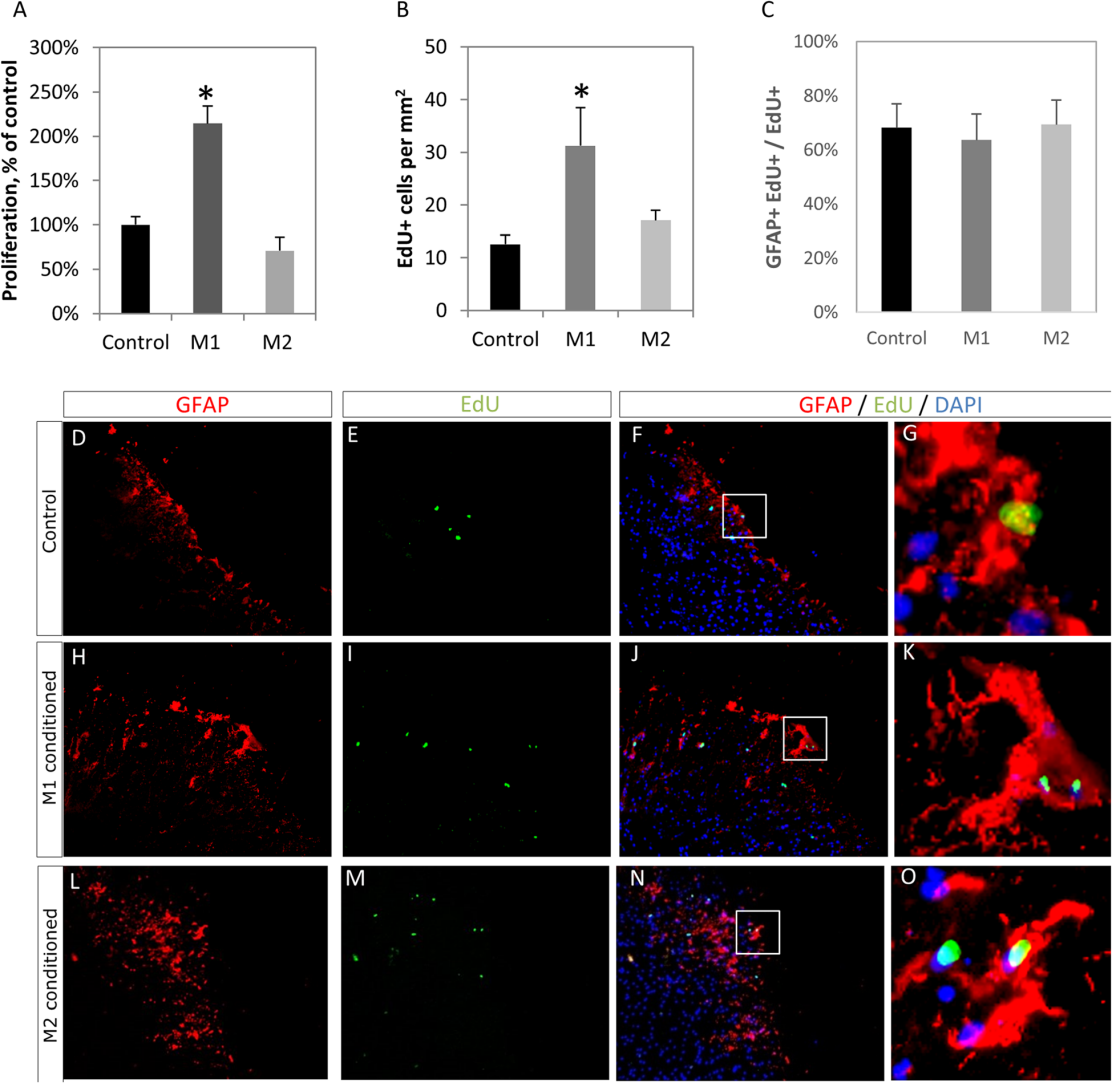
Effects of macrophage-derived soluble factors on spinal cord organotypic cultures. **a** Whole-slice MTT shows increased cellular respiration following incubation with M1-conditioned, but not M2-conditioned medium. **b** Cord cultures incubated for 72 h with conditioned medium of M1, but not M2, macrophages show increased numbers of dividing cells, as shown by EdU nucleotide incorporation. **c** The proportion of EdU incorporating cells that also express GFAP is not changed in experimental conditions. **d**–**o** Immunohistochemistry on cryosectioned slice cultures shows a significant fraction of dividing cells are GFAP+ astrocytes

To investigate the effects of soluble factors from macrophages on the spinal cord, organotypic spinal cord cultures were incubated with M1 or M2 macrophage-conditioned medium for 72 h. M1- but not M2-stimulated cultures showed increased metabolic activity (*p* = 0.028), in MTT assays (Fig. 2a, *n* = 4 per group). Furthermore, a significantly higher (*p* = 0.016) density of EdU incorporating cells (Fig. 2b) was found in M1-stimulated cultures (*n* = 3 per group), compared to control or M2. When the identity of proliferating cells was investigated by combined EdU incorporation and immunohistochemistry on sectioned cultures, a significant fraction of EdU incorporating cells in all conditions were found to express the astrocyte marker GFAP (Fig. 2c–o), with percentages of 68 ± 8, 64 ± 9 and 69 ± 9 for control, M1 and M2, respectively.

### Primary astrocytes show a proliferative response to M1 macrophage soluble factors

**Fig. 3:**
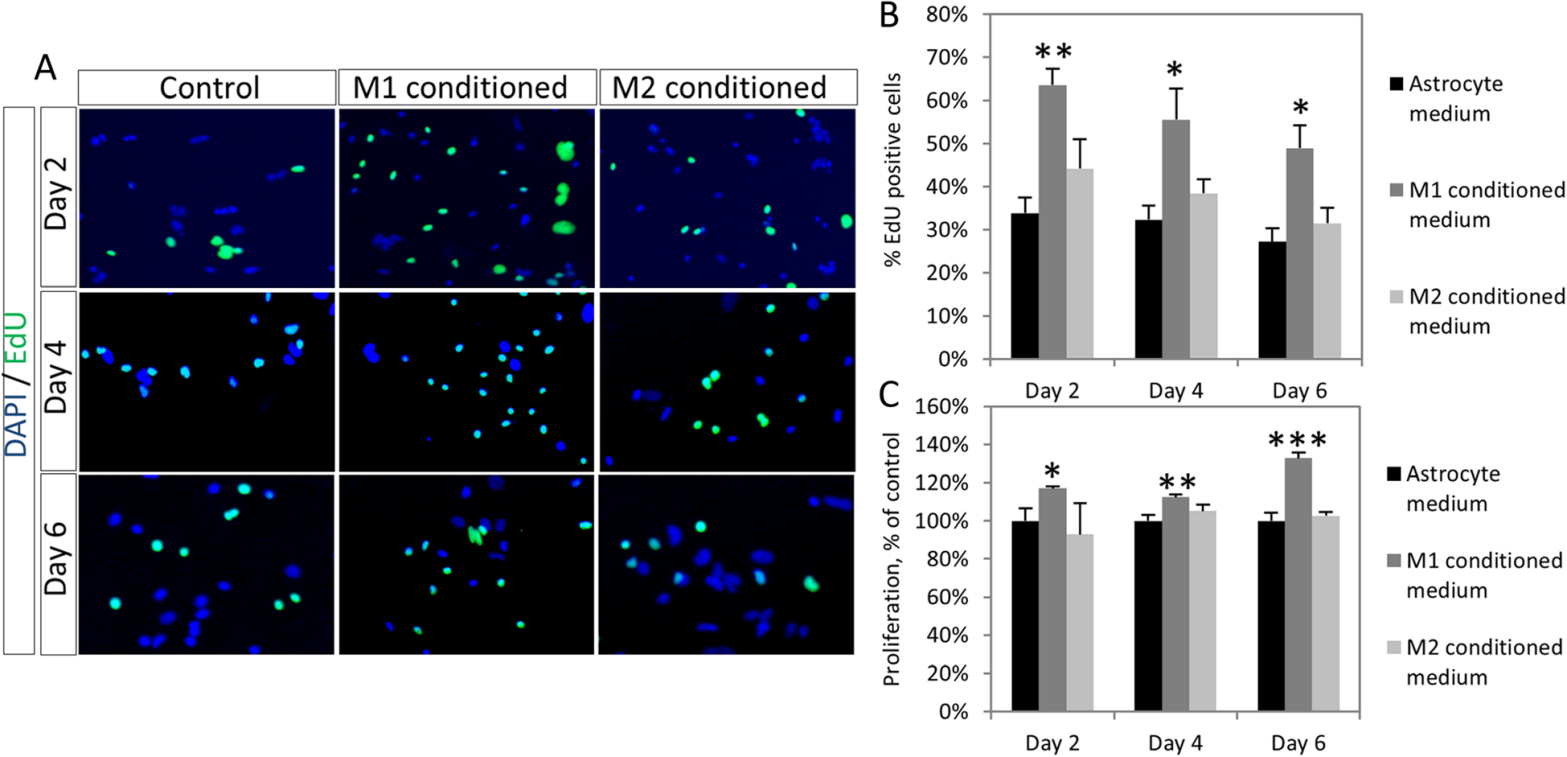
Effects of macrophage soluble factors on primary astrocytes. **a** Primary astrocytes incubated with control or macrophage-conditioned medium show high levels of cell division as seen by EdU incorporation. **b** Quantification of number of EdU+ cells shows a significantly higher percentage of cell division in M1- but not M2-stimulated cells at all time points. **c** MTT assays on macrophage-stimulated astrocytes again show a proliferative effect of M1-, but not M2-, conditioned medium

In order to further confirm the proliferative effects of M1 macrophage soluble factors, primary astrocytes were used. Incubation of astrocytes with both M1 and M2 macrophage-conditioned medium for 2, 4 or 6 days (*n* = 4) clearly shows a significant increase in the proportion of EdU incorporating cells, with *p* = 0.005, *p* = 0.022 and *p* = 0.012 for days 2, 4 and 6, respectively (Fig. 3a, b). This result was confirmed through MTT assays, with again significantly higher metabolic activity at days 2, 4 and 6, with *p* = 0.032, *p* = 0.009 and *p* < 0.001, respectively.

### M1 and M2 macrophages have opposite effects on astrocyte reactivity

**Fig. 4:**
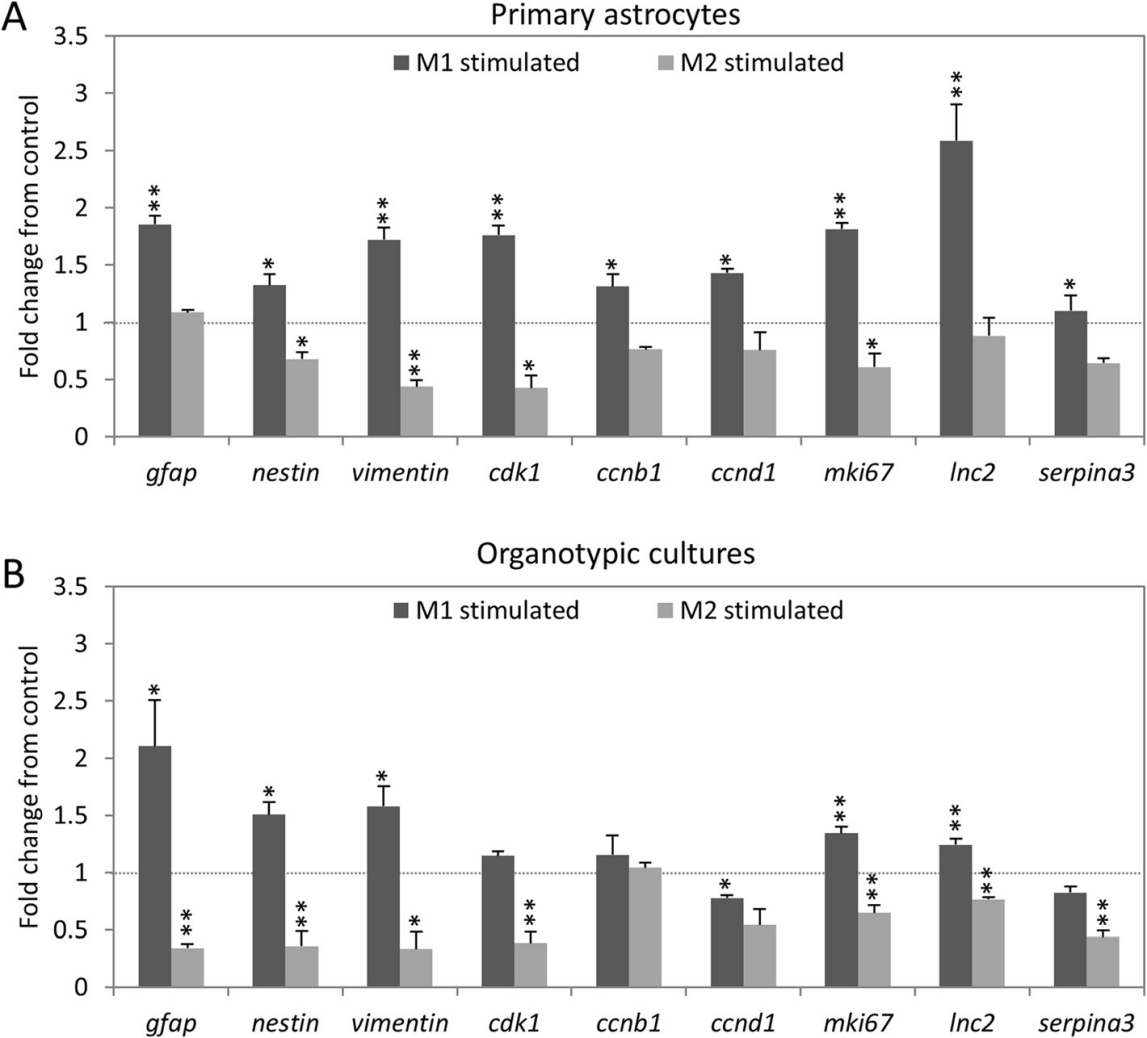
M1 and M2 macrophage effects on astrocyte reactive expression pattern. **a** In primary astrocytes, qPCR for gene expression associated with a reactive astrocyte phenotype shows opposite effects following stimulation with M1 or M2 macrophage-conditioned medium. Expression of most genes is upregulated following M1 stimulation and downregulated following M2 stimulation. **b** In spinal cord organotypic cultures, very similar gene expression changes are seen

The reactive marker expression of primary astrocytes was investigated by qPCR for genes known to be strongly upregulated in reactive astrocytes [26]. In primary macrophages, all genes investigated, excluding *serpinA3*, were significantly upregulated compared to control following M1 stimulation, and five out of nine investigated genes (*nestin, vimentin, cdk1, mki67 and serpinA3*) were significantly downregulated following M2 stimulation (Fig. 4a). In organotypic spinal cord cultures (Fig. 4b), gene expression is similar, with all genes except *cdk1* and *ccnb1* being significantly upregulated following M1 stimulation and all genes apart from *ccnb1* being downregulated following M2 stimulation.

### Previously stimulated astrocytes can modulate macrophage proliferation and TNFα production

**Fig. 5:**
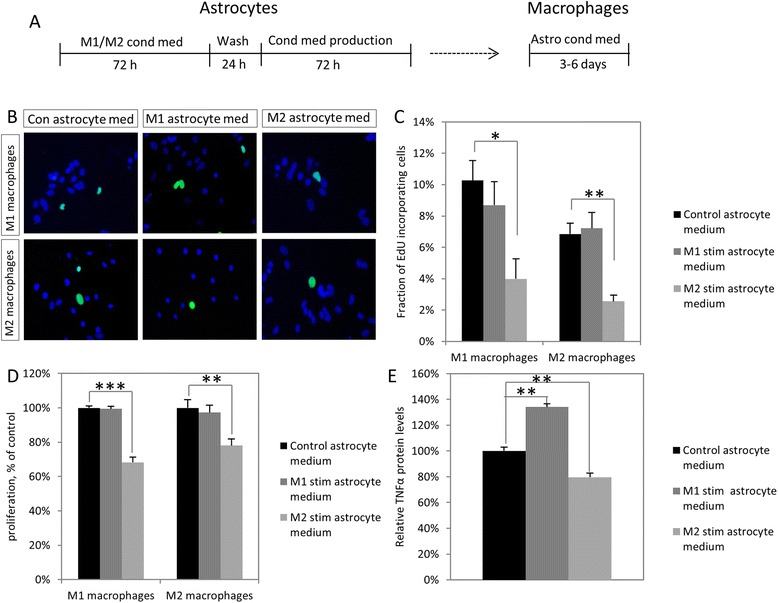
Effects of soluble factors from previously stimulated astrocytes on primary macrophages. **a** Experimental set-up. Astrocytes were stimulated with conditioned medium from M1 or M2 macrophages or left unstimulated. Following a wash, conditioned medium was then collected from these and incubated with fresh primary macrophages. **b**, **c** EdU incorporation assays show medium from astrocytes previously stimulated with M2-conditioned medium has an anti-proliferative effect on both M1 and M2 macrophages following 3 days of incubation. **d** The anti-proliferative effect is confirmed using MTT assays. **c** As with M1 macrophages, the proliferation of M2 macrophages is also lower when incubated with M2-stimulated astrocyte medium. **e** In M1 macrophages, TNFα release following β-glucan stimulation, as measured by ELISA, is also affected, with significantly higher release following incubations with M1-stimulated astrocyte medium, and a significant decrease after incubation with M2-stimulated astrocytes medium

In order to investigate whether any feedback exists from astrocytes back to macrophages, a two-step experiment was performed. Astrocytes were stimulated with M1 or M2 macrophage-derived conditioned medium, after which conditioned medium from these stimulated astrocytes was collected, which was then added to fresh M1 or M2 macrophages for EdU incorporation assays (Fig. 5b, c). Conditioned medium from astrocytes previously stimulated with M2-conditioned medium significantly decreased the mitotic index in both M1 (*p* = 0.017) and M2 macrophages (*p* = 0.007). Again, this effect was confirmed by MTT assays (*n* = 4, Fig. 5d), with a significant reduction in metabolism in both M1 (*p* < 0.001) and M2 (*p* = 0.009) macrophages. Apart from proliferation, β-glucan-induced TNFα production by M1 macrophages is also affected, with medium from previously M1-stimulated astrocytes able to significantly (*p* < 0.001) increase production, whereas M2-stimulated astrocyte medium decreases production (*p* = 0.004). There was no effect on M2 macrophages (data not shown).

## Discussion

Given the current poor prognosis for functional recovery following SCI, a thorough and full understanding of the processes during that recovery period is crucial for improving treatments. Here, we have studied the interplay between infiltrating macrophages and astrocytes. In our study, we focused on a moderate contusive injury and assessed macrophage response at the crucial time point of the onset of glial scar formation. We observed a mixed M1/M2 response, with a higher proportion of M2 macrophages. Although this seems to be in contradiction with some reports suggesting the response is predominantly M1, the ratio is most likely dependant on both the severity and nature of the injury and the time point investigated.

In accordance with our hypothesis that glial proliferation in the cord following injury may be driven by macrophage infiltration, spinal cord organotypic cultures exposed to conditioned medium from pro-inflammatory M1 macrophages showed increased glial proliferation, which was further confirmed though the use of primary astrocytes. Apart from proliferation, soluble factors from M1 also induce a reactive phenotype in both isolated astrocytes and organotypic cultures, with M2 factors having the opposite effect. This shows that M1 macrophages can promote both hallmarks of reactive astrogliosis, astrocyte proliferation and induction of specific gene expression patterns. The exact mechanisms responsible for this remain to be identified.

**Fig. 6:**
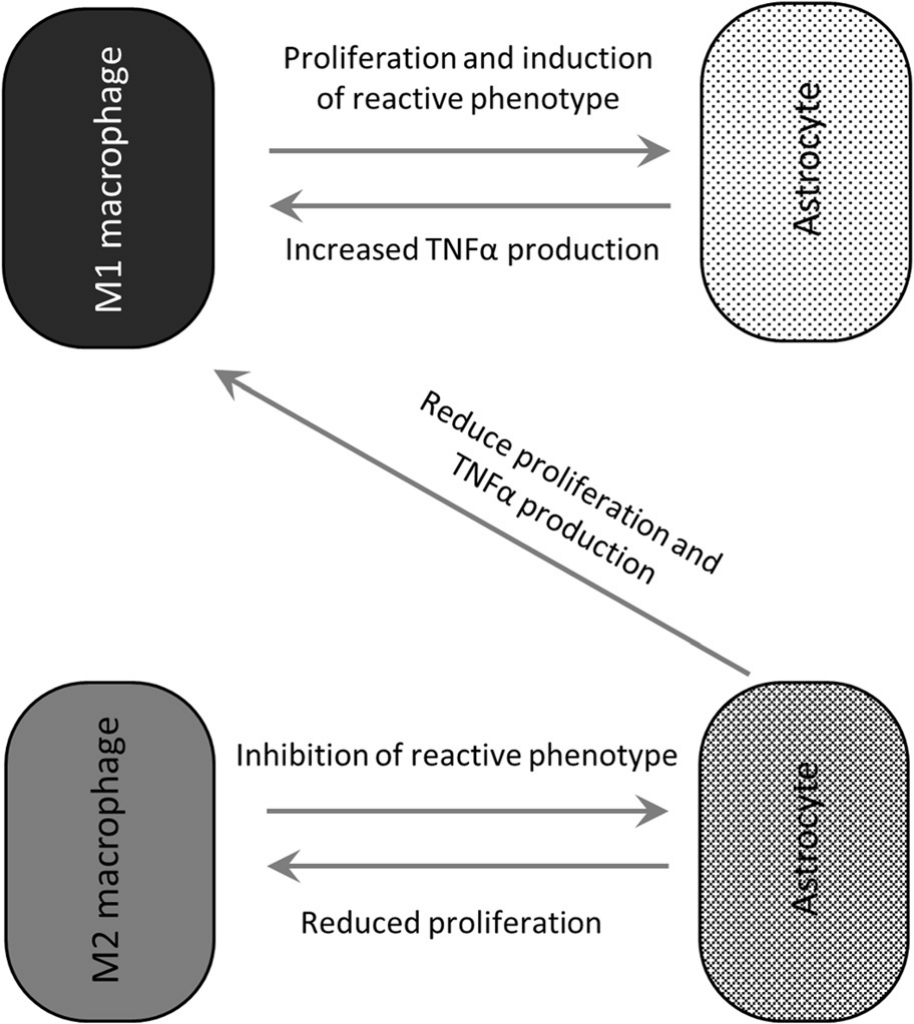
Schematic representation of astrocyte-macrophage interactions. M1 macrophages produce factors that induce astrocyte proliferation and reactive phenotype. These astrocytes are then able to increase TNFα expression on M1 macrophages. M2 macrophages produce factors that inhibit reactive astrocyte gene expression. Stimulated astrocytes can reduce the proliferation of both M1 and M2 macrophages and can dampen TNFα production in M1 macrophages

The fact that M2-stimulated astrocytes produce factors that can significantly decrease proliferation of both M1 and M2 macrophages, and modulate TNFα production, indicates the likely existence of a negative feedback loop to control the inflammatory response following SCI. Although the presence of M1 macrophages in the injury site will drive reactive gliosis, the large numbers of M2 macrophages also present will counteract this effect, both through decreasing astrocyte reactivity and through decreasing macrophage proliferation via astrocytes. The factor(s) mediating this feedback are as yet not known. A link between astrocytes and immunosuppression in SCI has been made before [18], and astrocytes may also limit the neuroinflammation following cerebral stroke, in a TGFβ1-dependent mechanism [27]. However, it must also be noted that TGFβ1 has been implicated in promoting glial scar formation [21], again indicating the double-edged sword of the immune response. The interactions between astrocytes and macrophages are schematically summarised in Fig. 6.

Although this study strongly suggests that M1 macrophages play an important role in the development of astrogliosis, the efficacy of immunosuppressive therapy through steroid treatment in SCI has been under debate. This illustrates the fine balance between the pro-inflammatory and gliosis-inducing M1 macrophages and the dampening role of the M2 macrophages and drives home the need for more targeted therapies, aimed at limiting the M1 response. Achieving this will likely be a great help in SCI treatment, and, possibly in conjunction with one of the many cell transplantation strategies currently under investigation, can hopefully lead to a better functional outcome for patients.

## Additional file

Additional file 1: Table S1. Sequences and product sizes for reactive astrocyte genes investigated by qPCR.
