# Supplementary material for: Crosstalk between macrophages and astrocytes affects proliferation, reactive phenotype and inflammatory response, suggesting a role during reactive gliosis following spinal cord injury
Source: J Neuroinflammation. 2015 May 30;12:109. doi: 10.1186/s12974-015-0327-3 (PMC4457974; doi:10.1186/s12974-015-0327-3)
Supplement: Additional file 1: Table S1. — Sequences and product sizes for reactive astrocyte genes investigated by qPCR. [file 12974_2015_327_MOESM1_ESM.docx]

**Supplementary data**

| Table S1: Sequences and product sizes for reactive astrocyte genes investigated by qPCR | | |
| --- | --- | --- |
| Gene | Sequences | Product size (bp) |
| *gfap* | 5’-GCTCCAAGATGAAACCAACCTG | 124 |
|  | 5’-CTCCTCCAGCGATTCAACCTT |  |
| *nestin* | 5’-GTCCCTTAGTCTGGAAGTGGC | 112 |
|  | 5’-CTTGGGGTCAGGAAAGCCAA |  |
| *vimentin* | 5’-GCGAGAGAAATTGCAGGAGGA | 111 |
|  | 5’-CGTTCAAGGTCAAGACGTGC |  |
| *cdk1* | 5’-ACAGAGAGGGTCCGTCGTAA | 115 |
|  | 5’-GCCAGTGACTCTGTGTCTACC |  |
| *ccnb1* | 5’-ACAACGGTGAATGGACACCA | 113 |
|  | 5’-GAGGCCACAGTTCACCATGA |  |
| *ccnb1* | 5’-CCATGCTCAAGACGGAGGAG | 117 |
|  | 5’-CACAGACCTCCAGCATCCAG |  |
| *mki67* | 5’-GGACCATGGCGTCCTCG | 105 |
|  | 5’-CAATACTCCTTCCAAACAGGCAG |  |
| *lcn2* | 5’-TGTCACCTCCATCCTGGTCA | 124 |
|  | 5’-CTGTACCTGAGGATACCTGTGC |  |
| *serpina3* | 5’-CTCTCAGGTGGTCCACAAGG | 107 |
|  | 5’-GAGGGTACAGTTTCGCAGACA |  |
| *gapdh* | 5’-AGGTCGGTGTGAACGGATTTG | 123 |
|  | 5’-TGTAGACCATGTAGTTGAGGTCA |  |
